# A comparative study of identical VMAT plans with and without jaw tracking technique

**DOI:** 10.1120/jacmp.v17i5.6252

**Published:** 2016-09-08

**Authors:** Hao Wu, Fan Jiang, Haizhen Yue, Qiaoqiao Hu, Jian Zhang, Zhuolun Liu, Jian Gong, Sha Li, Jianhao Geng, Yibao Zhang

**Affiliations:** ^1^ Key Laboratory of Carcinogenesis and Translational Research (Ministry of Education/ Beijing) Department of Radiation Oncology Peking University Cancer Hospital & Institute Beijing China

**Keywords:** jaw tracking, VMAT, MLC transmission, treatment planning, optimization

## Abstract

The unwanted radiation transmission through the multileaf collimators could be reduced by the jaw tracking technique which is commercially available on Varian TrueBeam accelerators. On the basis of identical plans, this study aims to investigate the dosimetric impact of jaw tracking on the volumetric‐modulated arc therapy (VMAT) plans. Using Eclipse treatment planning system (TPS), 40 jaw‐tracking VMAT plans with various tumor volumes and shapes were optimized. Fixed jaw plans were created by editing the jaw coordinates of the jaw‐tracking plans while other parameters were identical. The deliverability of this artificial modification was verified using COMPASS system via three‐dimentional gamma analysis between the measurement‐based reconstruction and the TPS‐calculated dose distribution. Dosimetric parameters of dose‐volume histogram (DVH) were compared to assess the improvement of dose sparing for organs at risk (OARs) in jaw‐tracking plans. COMPASS measurements demonstrated that over 96.9% of structure volumes achieved gamma values less than 1.00 at criteria of 3 mm/3%. The reduction magnitudes of maximum and mean dose to various OARs ranged between 0.06%∼6.76%(0.04∼7.29 Gy) and 0.09%∼7.81%(0.02∼2.78 Gy), respectively, using jaw tracking, agreeing with the disparities of radiological characteristics between MLC and jaws. Jaw tracking does not change the delivery efficiency and total monitor units. The dosimetric comparison of VMAT plans with and without jaw tracking confirms the physics hypotheses that reduced transmission through tracking jaws will reduce doses to OARs without sacrificing the target dose coverage because it is meant to be covered by radiation beams going through the opening.

PACS number(s): 87.55.de, 87.55.dk

## I. INTRODUCTION

The multileaf collimator (MLC) is essential for modern radiotherapy techniques including three‐dimentional conformal radiotherapy,^(1)^ intensity‐modulated radiotherapy (IMRT),^(2)^ and volumetric‐modulated arc radiotherapy (VMAT).^(3)^ As a tertiary collimator mounted under the jaws (secondary collimator) of Varian accelerators, the combination of jaws and MLC allows transmission less than 0.1% of the original intensity.^(4)^ However, for various jaw sizes covered only by MLC, the transmitted dose rate could be 0.90%‐4.40% (6 MV photon) or 1.14%‐7.00% (18 MV) higher than that shielded by jaws or both MLC and jaws.^5^, ^6^ These inherent differences enabled potential better sparing of organs at risk (OARs) from transmitted radiation through MLC either by manual adjustment of jaw positions,^(7)^ or by jaw‐tracking technique which continuously adjusting the main jaws to tangentially enclose the distal apertures shaped by MLC.

The benefits of jaw tracking have been well assessed for IMRT based on the same plan except for the jaw patterns,^8^, ^9^, ^10^, ^11^ However, bearing more variables than IMRT, such as gantry rotation speed, dose rate variety, jaw translation direction, and speed and range, any reoptimization with/without jaw tracking (even with the same optimization objectives) can change these parameters and the MLC sequences,^(12)^ hence the interplan differences were not exclusively induced by the jaw tracking technique^13^, ^14^ Alternatively, the artificial conversion from VMAT to jaw‐tracking static arc plans failed to include jaw tracking into the optimization procedure, hence the suboptimal plans may violate the mechanical constraints of jaws and were clinically not deliverable by the accelerators.^(15)^ Therefore, the dosimetric impact ofjaw tracking has not been evaluated based on the same VMAT plans by far, which is the aim of this study.

## II. MATERIALS AND METHODS

### A. VMAT plan creation

Using Varian Eclipse TPS (Version 11.0; Varian Medical Systems, Palo Alto, CA), jaw‐tracking VMAT plans were optimized for 10 head and neck (nasopharynx), 10 thoracic (lung), 10 abdominal (gastric), and 10 pelvic (cervical) cancer patients, respectively, using 6 MV photon for head and neck, 8 MV photon for thoracic, and 10 MV for the other patients. Although the settings for field size and collimator rotation were highly planner‐dependent, they were mainly determined by the observation from beam's eye view to strike a balance amongst the considerations of target coverage, OAR sparing, and mechanical limitations of MLC (motion speed and range). The isocenter of PTV was calculated by TPS, and minor modifications were involved when necessary (such as matching the decimals with the mechanical accuracy of treatment couch). The range of body was defined to ensure enough CT anatomy beyond PTV borders (≥5 cm) for peripheral dose calculation using anisotropic analytical algorithm (AAA, Version 11.0.31). Plans were executed on a Varian TrueBeam accelerator (Varian Medical Systems) equipped with Millennium 120 MLC system. The leaf transmission factors as configured for 6 MV, 8 MV, and 10 MV beams in the TPS were 1.37%, 1.56%, and 1.58%, respectively. The 40 plans involved various target sizes and shapes (the mean volume ±1 SD of PTV were 833±212, 387±179, 465±328, and $1684\pm 182\;{\text{cm}}^{3}$ for head and neck, thoracic, abdominal, and pelvic patients, respectively, can be found as Supplementary Material available at www.jacmp.org. A table showing staging of the selected cancer patients can be found in Appendix A. The dose prescription for each disease region was consistent (70/60 Gy SIB, 60 Gy, 50 Gy, and 60/45 Gy SIB, respectively) to make the absolute dose to OARs comparable. SIB indicates simultaneous‐integrated boosting.

In order to preserve all other plan parameters except for the jaw positions, the original jawtracking VMAT plan was duplicated and renamed as ‘fixed jaw plan’, and the four jaws were locked asymmetrically at their mostly retracted locations as recorded in the original jaw‐tracking plan by taking the following steps: By right clicking the ‘Field 1’ in the ‘Context Window’ on the left, selecting ‘Fit Collimator to Structure’, and pushing ‘Fit’, the jaw coordinates, as indicated in the ‘Fields’ tablet of ‘Info Window’ on the bottom, were activated for editing. Then the X1, X2, Y1, and Y2 jaws were retrieved to the positions as displayed in the original jaw‐tracking plan, same as their maximal values in the ‘Control Points’ tablet of the ‘Field Properties’ window. By repeating the aforementioned workflow for other fields (if any) and calculating the final doses, a fixed jaw plan was generated where the jaw pattern was the only modified parameter from the jaw‐tracking plan. The mechanical coherences and disparities were visually confirmed by inspecting the ‘Control Points’ of fields, the ‘Leaf Positions’ of MLC, and ‘Smart MLC Segment Animation’ on TPS.

### B. Plan execution and measurement based verification

To ensure the modification of jaw coordinates does not undermine the capability and accuracy of plan delivery, measurement based three‐dimensional dose reconstruction was compared with the TPS calculated distribution using COMPASS verification system (IBA Dosimetry, Schwarzenbruck, Germany). When the VMAT plans were executed, the commissioned MatriXX detector (IBA Dosimetry) and calibrated angle sensor were mounted to the TrueBeam accelerator for measurement. Using COMPASS software (version 3.1), the responses recorded by the MatriXX and the angles detected by the sensor were reconstructed into three‐dimensional dose distributions in patient CT anatomy. The agreement between the TPS calculation and measurement‐based reconstruction was compared by three‐dimensional gamma analysis (3 mm/3% criteria, reference dose max calculation type) and reported as the percentage volume with gamma smaller than 1.00 for a structure.

### C. Plan evaluations

Based on the DVH data exported in tabular format, the mean DVHs of 10 patients of the same region were calculated for plans with/without jaw tracking respectively using an in‐house MATLAB code (MathWorks, Natick, MA). SigmaPlot (Systat Software, Inc. San Jose, CA) was used to plot the mean DVHs with and without jaw tracking on the same figure to display the overall disparities. Numerically, the maximum and mean doses of each OAR were compared by paired /‐test to assess the impact of jaw tracking. Mean dose to the targets and the monitor units (MUs) were also assessed for each plan.

## III. RESULTS

### A. Plan verification

The TPS based visual inspection of jaw tracking and fixed jaw plans presented full coincidences including field control points (meter set weight, gantry rotation, and gantry speed) and MLC leaf positions (field size, bank A and B locations). The discrepancies of jaw patterns and the consistency of MLC translation between the paired plans were also explicitly displayed in the smart MLC segment animation. The animation example can be found as Supplementary Material available at www.jacmp.org.

COMPASS‐based, three‐dimensional gamma analysis demonstrated that the plans with arbitrarily modified jaws were well executable; the passing rates for the jaw tracking and fixed jaw plans were over 96.9% and 99.2%, respectively, for the tested cases. Most OARs achieved passing rate of 100%.

### B. Dosimetric comparison

The dosimetric advantages (%) achieved by jaw tracking (T) over fixed jaw (F) technique are listed in Table 1, in the form of $(F-T)/T^{*}100$. Due to the additional jaw shielding, dose reduction magnitudes of 0.06%∼6.76% and 0.09%∼7.81% were observed for the maximum and mean OAR dose respectively, and most (50/60=83.33%) of these reductions were statistically significant (p<0.05). Table 2 lists the corresponding statistics of the absolute dose changes (Gy). Reductions of maximum and mean dose to various OARs ranged between 0.04∼7.29 Gy and 0.02∼2.78 Gy, respectively.

**Figure 1:**
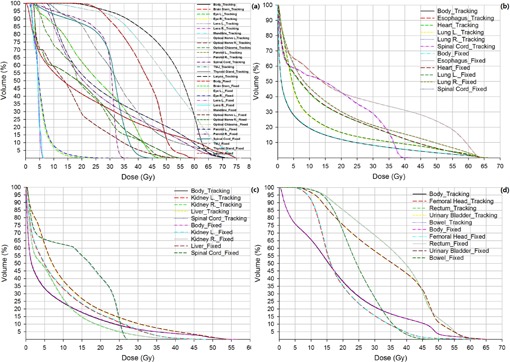
Comparison of mean dose‐volume histograms (DVHs) between jaw‐tracking and fixed jaw plans: head and neck (a), thoracic (b), abdominal (c), and pelvic (d) patients.

Analogously, Fig. 1 shows the macroscopic reduction of OAR exposure using jaw tracking in terms of the mean DVHs of head and neck (a), thoracic (b), abdominal (c), and pelvic patients (d), respectively. In addition to the statistics in 1, 2, Fig. 1 also displays the jaw trackinginduced reductions of other parameters of clinical concern, such as lung $V_{5\text{Gy}}$ (by 1.57% and 0.79% for left and right), $V_{20\text{Gy}}$ (by 0.17% and 0.27% for left and right), and $V_{30\text{Gy}}$ (by 0.11% and 0.15% for left and right); heart $V_{30\text{Gy}}$ and $V_{40\text{Gy}}$ (by 0.13% and 0.11%); rectum $V_{40\text{Gy}}$ and $V_{50\text{Gy}}$ (by 0.13% and 0.01%); bladder $V_{40\text{Gy}}$ and $V_{50\text{Gy}}$ (by 0.15% and 0.11%), respectively.

Artificially locking the jaws did not change the MU (hence did not impact the delivery efficiency), yet increased the target mean dose by 0.08%, 0.12%, 0.15%, and 0.09% for head and neck, thoracic, abdominal, and pelvic groups, respectively.

**Table 1:** Relative dose reduction $\left(\Delta =(\text{Fixed}-\text{Tracking})/{\text{Tracking}}^{*}100\%\right)$ to the OARs using jaw‐tracking technique than the fixed jaw plans.

	$\Delta_{\text{max}}(\%)$			$\Delta_{\text{mean}}(\%)$		
	Mean±SD	*95%CI*	P	Mean±SD	*95%CI*	P
Body	0.07±0.05	0.04∼0.11	0.00	0.50±0.21	0.35∼0.65	0.00
Larynx	0.11±0.05	0.07∼0.14	0.00	0.14±0.06	0.09∼0.18	0.00
Brain Stem	0.13±0.05	0.10∼0.16	0.00	0.89±0.74	0.36∼1.42	0.00
Eye L.	1.61±1.45	0.57∼2.65	0.00	6.22±4.44	3.04∼9.39	0.00
Eye R.	2.82±4.34	−0.29∼5.92	0.03	5.91±5.22	2.18∼9.64	0.00
Lens L.	6.76±4.89	3.26∼10.26	0.00	7.81±5.68	3.75∼11.88	0.00
Lens R.	6.40±5.08	2.76∼10.04	0.00	6.51±4.98	2.94∼10.07	0.00
Mandible	0.06±0.04	0.03∼0.09	0.00	0.16±0.04	0.13∼0.18	0.00
Optical N. L.	3.63±9.27	−3.00∼10.26	0.12	4.21±7.53	−1.18∼9.60	0.01
Optical N. R.	2.21±4.77	−1.20∼5.62	0.04	3.17±5.17	−0.53∼6.87	0.00
Optical Chiasma	6.32±17.09	−6.81∼19.46	0.19	3.43±7.23	−2.13∼8.98	0.03
Parotid L.	0.07±0.04	0.04∼0.09	0.00	0.28±0.13	0.19∼0.38	0.00
Parotid R.	0.07±0.03	0.05∼0.09	0.00	0.40±0.29	0.18∼0.62	0.00
Spinal Cord	0.20±0.08	0.14∼0.26	0.00	0.23±0.11	0.15∼0.31	0.00
TMJ	0.07±0.05	0.03∼0.11	0.01	0.30±0.28	0.10∼0.50	0.00
Thyroid Gland	0.06±0.04	0.03∼0.09	0.00	0.09±0.06	0.05∼0.13	0.00
Body	0.10±0.11	0.02∼0.18	0.02	1.22±0.69	0.74∼1.72	0.00
Esophagus	0.09±0.10	0.02∼0.17	0.02	0.62±0.82	0.04∼1.21	0.00
Heart	0.23±0.34	−0.01∼0.47	0.01	1.82±1.91	0.45∼3.18	0.00
Lung L.	0.71±0.93	0.04∼1.37	0.05	2.31±2.01	0.87∼3.75	0.00
Lung R.	0.10±0.13	0.01∼0.19	0.03	1.17±0.87	0.55∼1.79	0.00
Spinal Cord	1.41±2.12	−0.10∼2.93	0.07	1.24±0.94	0.57∼1.92	0.00
Body	0.16±0.16	0.04∼0.27	0.01	1.14±1.34	0.18∼2.10	0.04
Kidney L.	0.33±0.50	−0.14∼0.79	0.18	1.52±1.32	0.30∼2.74	0.06
Kidney R.	0.34±0.33	0.06∼0.62	0.01	1.38±1.48	0.15∼2.62	0.04
Liver	0.13±0.16	0.02∼0.25	0.03	0.85±0.63	0.40∼1.30	0.01
Spinal Cord	2.20±3.56	−0.34∼4.75	0.08	1.38±1.52	0.29∼2.47	0.01
Body	0.08±0.05	0.04∼0.11	0.00	0.50±0.19	0.36∼0.64	0.00
Femoral Head	0.13±0.06	0.09∼0.17	0.12	0.65±0.29	0.44∼0.86	0.04
Rectum	0.11±0.04	0.08∼0.14	0.00	0.18±0.09	0.12∼0.25	0.18
Urinary Bladder	0.10±0.06	0.05∼0.14	0.64	0.17±0.07	0.11∼0.22	0.00
Bowel	0.09±0.06	0.05∼0.14	0.00	0.10±0.05	0.06∼0.13	0.00

$\Delta D_{\text{max}}=\text{relative difference of maximum dose}$; $\Delta D_{\text{mean}}=\text{relative difference of mean dose}$; SD=standard deviation; CI=confidence interval; L.=left; R.=right; N.=nerve; TMJ=temporomandibular joint.

**Table 2:** Absolute maximum and mean dose to critical structures in jaw‐tracking and fixed jaw plans. Dose was reported as mean ±1SD.

	*Maximum Dose (Gy)*			*Mean Dose (Gy)*		
	*Tracking*	*Fixed*	Δ	*Tracking*	*Fixed*	Δ
Body	76.54±0.71	76.60±0.72	0.06	22.23±2.36	22.34±2.37	0.11
Larynx	54.83±4.37	54.89±4.38	0.06	41.97±6.27	42.03±6.29	0.06
Brain Stem	47.98±2.64	48.04±2.64	0.06	25.52±4.70	25.73±4.64	0.21
Eye L.	16.25±7.42	16.48±7.47	0.23	5.01±1.74	5.32±1.84	0.31
Eye R.	17.48±9.06	17.83±8.98	0.34	5.21±2.03	5.50±2.07	0.29
Lens L.	4.43±1.15	4.73±1.23	0.30	3.87±0.96	4.18±1.07	0.31
Lens R.	4.34±1.32	4.62±1.37	0.27	3.85±1.15	4.09±1.20	0.25
Mandible	74.46±2.58	74.50±2.58	0.04	50.00±5.40	50.08±5.41	0.08
Optical N. L.	27.68±21.65	27.88±21.51	0.20	15.52±11.53	15.81±11.47	0.29
Optical N. R.	32.17±23.87	32.32±23.78	0.15	19.60±14.64	19.83±14.57	0.22
Optical Chiasma	29.52±16.99	29.93±16.52	0.42	20.87±12.73	21.11±12.59	0.24
Parotid L.	70.65±2.19	70.69±2.17	0.05	29.16±4.89	29.24±4.88	0.08
Parotid R.	69.30±4.22	69.34±4.22	0.05	26.93±4.43	27.03±4.40	0.10
Spinal Cord	34.69±0.99	34.76±1.01	0.07	28.11±1.60	28.18±1.61	0.06
TMJ	63.10±7.14	63.14±7.13	0.04	33.85±10.10	33.93±10.07	0.08
Thyroid Gland	69.70±3.36	69.75±3.38	0.04	52.28±2.95	52.32±2.94	0.05
Body	65.73±0.70	65.80±0.74	0.07	7.20±1.75	7.29±1.74	0.08
Esophagus	62.32±4.17	62.38±4.20	0.06	23.80±12.18	23.89±12.16	0.10
Heart	57.43±14.90	57.52±14.82	0.09	14.64±10.11	14.77±10.15	0.13
Lung L.	47.87±18.33	48.18±18.35	0.31	10.97±8.88	11.13±8.91	0.17
Lung R.	64.19±2.92	64.25±2.96	0.07	16.50±7.43	16.65±7.41	0.15
Spinal Cord	37.65±4.03	38.17±4.08	0.52	16.12±8.14	16.27±8.11	0.15
Body	54.07±1.19	54.15±1.17	0.08	7.36±3.02	7.45±3.08	0.10
Kidney L.	34.53±17.41	34.64±17.45	0.12	9.15±5.31	9.32±5.45	0.17
Kidney R.	30.80±12.45	30.88±12.45	0.08	7.11±3.87	7.22±3.94	0.11
Liver	53.30±1.13	53.37±1.14	0.07	11.67±3.12	11.78±3.19	0.11
Spinal Cord	23.42±9.22	24.05±9.69	0.63	13.50±8.11	13.70±8.21	0.20
Body	64.61±0.79	64.65±0.81	0.05	18.81±3.01	18.90±3.00	0.09
Femoral Head	46.32±5.23	52.52±7.16	6.20	18.14±1.58	20.93±2.34	2.78
Rectum	54.01±5.40	61.30±2.24	7.29	35.63±3.43	37.59±3.37	1.97
Urinary Bladder	59.59±4.68	60.76±5.76	1.17	32.77±4.05	34.89±3.35	2.11
Bowel	47.44±2.79	47.48±2.79	0.04	25.07±2.24	25.09±2.24	0.02

Δ=dose difference between jaw tracking and fixed jaw plans (Gy); L.=left; R.=right; N.=nerve; TMJ=temporomandibular joint.

## IV. DISCUSSION

Using the proposed approach to lock the jaws in the duplicated jaw‐tracking VMAT plans, the fixed jaw plans completely preserved the mechanical parameters as were optimized in the corresponding jaw‐tracking plans, except for the jaw coordinates. The deliverability of the arbitrarily modified plan was verified by COMPASS measurement and three‐dimensional dose reconstruction. The planed dose distributions in patient anatomy were clinically achievable by TrueBeam accelerator, without violating any physical constraints.

Agreeing with earlier reports,^1^, ^9^, ^10^, ^15^ jaw tracking exhibits superior OAR protection than fixed jaw technique for all anatomical regions and tumor shapes, without sacrificing delivery efficiency of VMAT plans. By limiting the jaws as the only variable, the magnitudes of OAR dose reduction observed in this study are generally consistent with the theoretical predictions from the dosimetric disparities of jaws and MLCs,^6^, ^8^, ^9^, ^15^ yet more diverse due to the complexities of clinical situations and tumor anatomies. While under a simplified condition, such as for the points under shielding during the whole treatment, the peripheral dose may not be affected significantly.^(16)^

Contrary to Schmidhalter's theoretical computations of increased PTV dose using jawtracking,^(9)^ we observed decreased doses to both the target and OARs in jaw‐tracking plans, agreeing with other investigators.^8^, ^15^ The target dose reduction can be ascribed to three facts: 1) To create inhomogeneous intensities, a large portion of target volume is shielded by MLC much of the time, where the transmitted radiation is modeled by TPS and incorporated into target dose, but largely eliminated by jaw tracking;^(17)^ 2) The shrunk field sizes, as defined by the jaws, lead to a smaller output factor, which is constantly larger in the fixed jaw plans; 3) Jaw tracking provides better shielding of surrounding OARs, which also reduces the scattered dose to the target from adjacent normal tissues.

Based on previous knowledge and dosimetric comparisons of this study, the advantages of jaw tracking are more appreciable in the following situations and clinical applications:

1. Dispersed multiple targets,^(11)^ or large PTV with irregular shape which varies dramatically from different beam's eye view (BEV),^(15)^ allowing considerable magnitude of jaw adjustment during the gantry rotation.^(16)^ The large variety of the reduction ranges indicated great diversity of tumor shapes and sizes in our population.
2. Critical structures of special concern (such as lenses, gonads, red bone marrow, $V_{5\text{Gy}}$ of lungs, and neural stem cells)^(18)^ can get extra shielding from jaw tracking at some BEV orientations. It can be observed from 1, 2 that relatively larger reduction in percentage were achievable for the OARs receiving lower absolute dose.
3. Plans with high MUs and long delivery time allowing more transmitted radiation through MLC.
4. Local relapse treated with radiotherapy previously, demanding more stringent constraints for OAR protection.
5. Pediatric patients with expectation of a long life span and higher risk of radiogenic secondary cancer.^19^, ^20^, ^21^
6. Plans utilizing higher photon energies where more radiation can penetrate through MLC which could be well blocked by the jaws.^(5)^

As for the limitations, artificially locking the jaw coordinates of the jaw‐tracking VMAT plans is not intended for clinical implementation but for the evaluation of jaw‐tracking technique on the basis of identical mechanical parameters only. Because the fixed jaw plans were not optimized using the same optimization objectives as the jaw‐tracking plans, the dosimetric findings may exaggerate the dose reduction; hence, all conclusions were drawn based on the identical fixed jaw VMAT plans. Plan optimization with jaw tracking enabled complies with the ‘ALARA’ principle and potentially maximizes the benefit to patients.

## V. CONCLUSIONS

By locking the jaw positions of the VMAT plans optimized with jaw tracking technique, this study investigated the dosimetric effects ofjaw tracking without changing other plan features. The reduction of OAR irradiation by jaw tracking not only lowers the risks of acute toxicity^(22)^ and secondary radiogenic cancer,^(20)^ but also enables potential target dose escalation as a tradeoff for a better tumor control.

## ACKNOWLEDGMENTS

This work was supported by National Natural Science Foundation of China (11505012, 81402535), Beijing Municipal Administration of Hospitals' Youth Programme (code: QML20151004) and Special Fund for Quality Scientific Research in the Public Welfare (201510001‐02). This study was approved by the Institutional Review Boards with waiver of informed consent.

## COPYRIGHT

This work is licensed under a Creative Commons Attribution 3.0 Unported License.

## Supporting information

Supplementary Material

Supplementary Material

Supplementary Material

Supplementary Material

Supplementary Material

## Appendix

**Table:**

Appendix A. Staging of the selected cancer patients.			
*Nasopharynx*	*Lung*	*Gastric*	*Cervical (FIGO)*
T3N2M0	T2aN1M0	T3N3aM0	Illb
T4N2M0	T4N3Mx	T4aN2M0	IIb
T3N1M0	T1N2M1	T4aN1M0	IIb‐IIIa
T3N2M0	T2N3M0	T4N+M0	Ib1
T4NxM0	T3N1M1	T4aN0M0	IIb
T3N1M0	T1N2M0	T4aN2M0	Ib1
T4bN20M0	T4N3M1	TxN+M1	IIIa
T3N2M0	T2N2M0	T4N1M0	IIIa
T3N2M0	T3N3Mx	T2N0M0	IIb
T3N2M0	T4N1M0	T3N3M0	IVb
