# Supplementary figures and images for: A comparative study of identical VMAT plans with and without jaw tracking technique
Source: J Appl Clin Med Phys. 2016 Sep 8;17(5):133–41. doi: 10.1120/jacmp.v17i5.6252 (PMC5874095; doi:10.1120/jacmp.v17i5.6252)

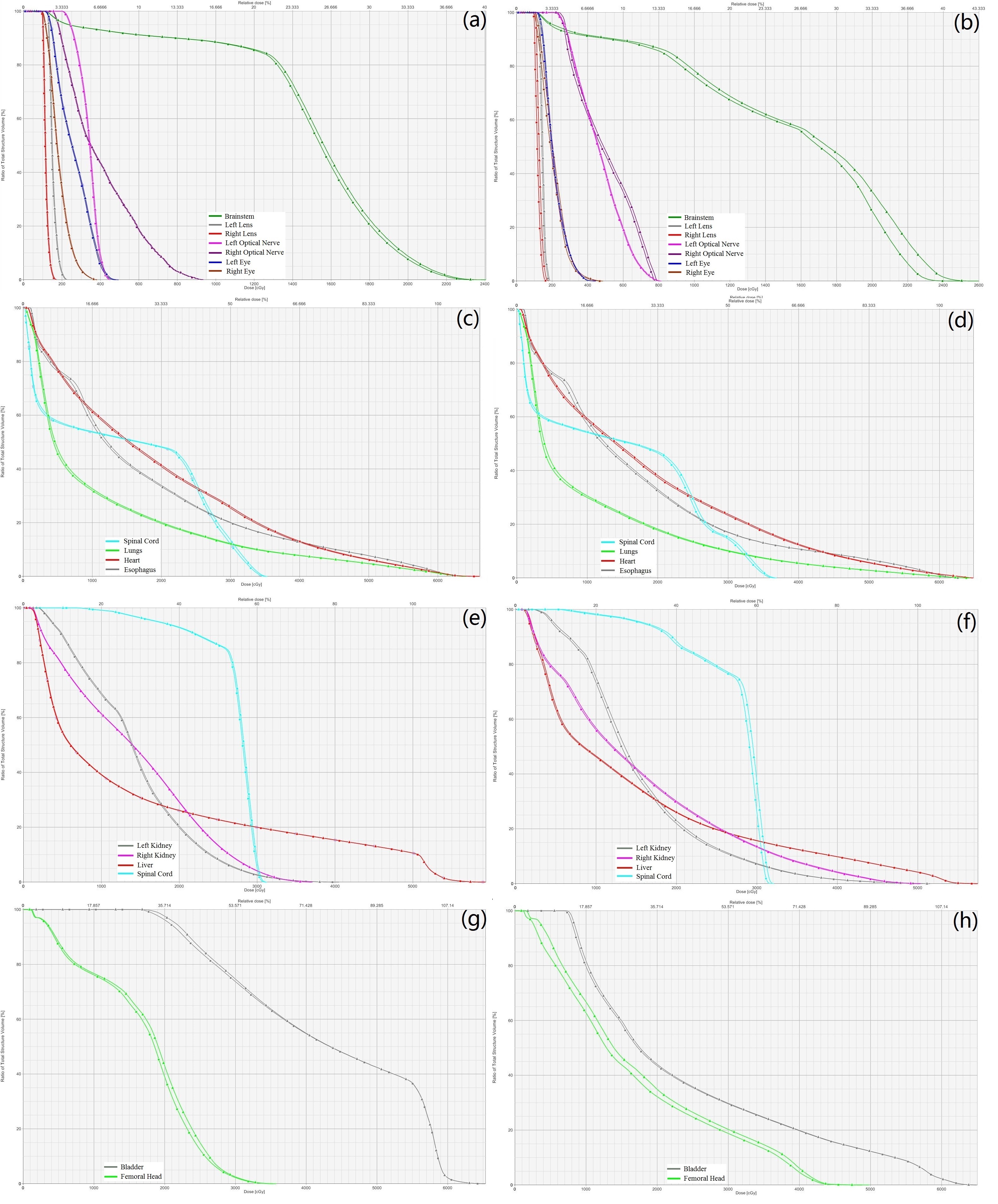

Supplement: Supplementary file 1 — Supplementary Material [file ACM2-17-133-s001.jpg]
