# Supplementary material for: A comparative study of identical VMAT plans with and without jaw tracking technique
Source: J Appl Clin Med Phys. 2016 Sep 8;17(5):133–41. doi: 10.1120/jacmp.v17i5.6252 (PMC5874095; doi:10.1120/jacmp.v17i5.6252)
Supplement: Supplementary file 5 — Supplementary Material [file ACM2-17-133-s005.docx]

Title Page

**A comparative study of identical VMAT plans with and without jaw tracking technique**

**Hao Wu^*1^, Fan Jiang^*1^, Haizhen Yue^1^, Jian Zhang^2^, Hui Zhang^2^, Qiaoqiao Hu^1^, Jian Zhang^1^, Zhuolun Liu^1^, Jian Gong^1^, Sha Li^1^, Yibao Zhang ^a1^**

*1.* *Key laboratory of Carcinogenesis and Translational Research (Ministry of Education/Beijing), Department of Radiation Oncology, Peking University Cancer Hospital & Institute, Beijing, 100142. China; 2. Division of Ionizing Radiation Metrology, National Institute of Metrology, Beijing, 100029. China*

*ybzhang77@gmail.com*

* Hao Wu and Fan Jiang contributed equally to this work.

^a^ Corresponding author: Yibao Zhang, Department of Radiation Oncology, Beijing Cancer Hospital, 52 Fucheng Road, Haidian, Beijing 100142 China; Phone: +8610-88196033; Fax: +8610-88196033; Email: ybzhang77@gmail.com

Running title: VMAT with/without jaw tracking
